# Applying the RE-AIM framework to evaluate the implementation of the Supporting and Enhancing NICU Sensory Experiences (SENSE) program

**DOI:** 10.1186/s12887-021-02594-3

**Published:** 2021-03-22

**Authors:** Roberta Pineda, Jessica Roussin, Jenny Kwon, Elizabeth Heiny, Graham Colditz, Joan Smith

**Affiliations:** 1 Chan Division of Occupational Science and Occupational Therapy, University of Southern California, Los Angeles, CA USA; 2 Department of Pediatrics, Keck School of Medicine, Los Angeles, CA USA; 3 Gehr Family Center for Health Systems Science and Innovation, University of Southern California, Los Angeles, CA USA; 4 Program in Occupational Therapy, Washington University, St. Louis, MO USA; 5 Department of Radiation Oncology, Washington University, St. Louis, MO USA; 6 Department of Therapy Services, St. Louis Children’s Hospital, St. Louis, MO USA; 7 Department of Surgery, Washington University, St. Louis, MO USA; 8 Department of Quality, Safety, and Practice Excellence, St. Louis Children’s Hospital, St. Louis, MO USA

**Keywords:** Parent, Environment, Therapy, Outcomes, Fidelity, Preterm, Reach, Effectiveness, Adoptability, Maintenance

## Abstract

**Background:**

To maximize the benefit of parent-directed, positive sensory exposures in the NICU, a structured sensory-based program titled the Supporting and Enhancing NICU Sensory Experiences (SENSE) program was developed that includes specific doses and targeted timing of evidence-based sensory exposures.

**Methods:**

The Reach, Effectiveness, Adoption, Implementation, Maintenance (RE-AIM) framework was used to systematically evaluate the SENSE program as an implementation strategy. One-hundred preterm infants ≤32 weeks gestation were studied (61 receiving the SENSE program and 39 standard-of-care). Parent education time and infant sensory exposures were tracked, and parents completed a questionnaire that probed their perceptions about the SENSE program.

**Results:**

One-hundered thirty-one families were recruited, and 100 (76%) enrolled. The SENSE program was initiated at an average postmenstrual age of 29.8 (±2.4) weeks; 4.9 (±5.6) days after birth. The average number of education sessions with families was 4.8 (±3.7) amounting to 72.3 (±37.4) total minutes over hospitalization. The total time of logged tactile and auditory exposures among SENSE recipients over the length of hospitalization was a median (IQ range) of 9325 (5295-15,694) minutes over an average of 10.1 (±7.6) weeks of hospitalization. There were differences in the proportion of tactile and auditory exposure targets received by the infant among those receiving the SENSE program compared to standard-of-care (91% compared to 48%; *p* < 0.0001). Ninety-five percent of infants tolerated the SENSE program as defined, with 5% of infants requiring intermittent adaptations or the interventions being stopped for a period that typically lasted 1–2 weeks. Earlier parent education was related to more parent participation in SENSE program interventions (*p* = 0.04). Eighty-five percent of participants receiving the SENSE program had most of the sensory interventions completed by parents, as opposed to the medical or sensory support team. Seventy-two percent of infants had at least 100% of the auditory and tactile doses conducted over the length of stay. Parents reported acceptability.

**Conclusion:**

The SENSE program had good reach, was effective and acceptable with minimal cost, was adopted, and had good fidelity. Insights from implementation of the SENSE program (within a research study) informed future strategies to aid maintenance during dissemination.

## Background

The evidence to support positive sensory exposures (such as massage, skin-to-skin care, or music) to improve outcomes for high-risk infants in the NICU and their families is well-understood [1–4]. However, differences in the use and interpretation of available evidence, as well as differences in parent education and empowerment in the NICU, are prevalent [5–11]. Subsequently, the consistent application of positive sensory exposures every day of hospitalization to optimize outcomes has not been achieved in many NICU settings [1, 12].

Due to the complex environment and differences in culture across NICUs, there is significant variability in the application of sensory-based interventions [12], reducing their benefit to the most vulnerable infants in the NICU. Therefore, an active plan to engage parents and/or surrogate caregivers to consistently provide positive, sensory-based interventions to their preterm infants throughout NICU hospitalization is critical. A program or implementation strategy, based on the best available evidence, is important to enable preterm infants to receive the full benefit of positive sensory experiences during the NICU stay.

The goal of the Supporting and Enhancing NICU Sensory Experiences (SENSE) program is to engage parents in consistently providing positive, developmentally-appropriate, evidence-based sensory interventions to their high-risk infants in the NICU every day of hospitalization. The SENSE program includes specific doses and targeted timing (based on postmenstrual age; PMA) of sensory exposures such as skin-to-skin care, infant massage, auditory exposure, holding, and rocking. The SENSE program was developed to optimize parent engagement, while maximizing daily positive sensory exposures to improve infant development and parent-infant interaction. A systematic and scientific process of development of the SENSE program was undertaken using a synthesis of research related to sensory interventions (known to improve outcome), parent and health care professional focus groups and interviews, and expert input [1, 12, 13]. While the SENSE program was developed to improve developmental outcomes based on current evidence, with doses for different sensory exposures defined prior to enrollment of participants for this study, future evidence may inform the need for changes to the SENSE program as new research emerges. The key features of the SENSE program are to engage parents in providing consistent, appropriate, positive sensory interventions across hospitalization. However, what those sensory exposures are, how much should be delivered, and at what intensity they should occur may change as new evidence emerges and program revisions are needed.

According to the Reach, Effectiveness, Adoption, Implementation, Maintenance (RE-AIM) framework, important components of an implementation strategy can be planned and evaluated [14]. Reach includes how well the intervention reached the target population, including their willingness to participate. Effectiveness refers to the impact of the intervention including negative responses, but also can include survey responses from the target population. Adoption is demonstrated by the willingness of people in the setting to initiate the program. Implementation includes fidelity of the intervention in the setting in addition to time and cost of the intervention, as well as the individual’s use of the intervention as defined. Maintenance refers to how well the intervention becomes part of routine practice at the setting (for > 6 months) [15]. These implementation outcomes enable empirical evaluation of implementation success in a real-world context by identifying contextual factors impacting the intervention [15, 16] and give insight into how successfully a new program can be implemented.

The aim of this study was to use a systematic approach to evaluate the SENSE program, as an implementation strategy, using the RE-AIM framework. We investigated the reach, effectiveness, adoption, and implementation of the SENSE program in a Level IV NICU, implemented as part of a research study. We hypothesized that the SENSE program could reach > 75% of those targeted, with < 10% attrition; that parent feedback would be largely positive; that parents would conduct the majority of the SENSE interventions and that total education time provided to parents would be < 2 h per infant, with no other associated costs when parents implement the sensory exposures themselves; and that 90% of the tactile and auditory exposures defined in the SENSE program would be carried out with preterm infants every day of hospitalization.

## Methods

This study was approved by the ethics committee/institutional review board of Washington University Human Research Protection Office, and parents signed informed consent.

### Participants

This study used a pre-defined sample to determine implementation outcomes, combining participants in a pilot study and a randomized clinical trial of the SENSE program. The sample size for the pilot study was defined as 30 parent-infant dyads to enable a large enough sample to permit refinement/optimization of educational materials. The sample size for the randomized clinical trial was defined as 70 parent-infant dyads, based on a power analysis of its primary outcome, the Communication score of the Ages and Stages Questionnaire at 1 year of age, while factoring anticipated attrition. Adequate sample size in implementation studies, such as this report, is difficult to pinpoint due to the evaluation of multiple factors that make up a complex system in health services research, many of the outcomes being descriptive in nature (see hypotheses), and not having a single outcome measure to run a power analysis on [17]. In our case, the sample size was predetermined based on use of participants from the overarching studies,

One-hundred thirty-one consecutive admissions of preterm infants born ≤32 weeks gestation and hospitalized in an 85-bed (expanded to 132-beds during the course of the study) Level IV NICU were recruited within 7 days of birth from October 2016 to June 2018. The Level IV NICU was in a free-standing children’s hospital that was part of an academic center. It was in an urban area in the Midwest. Infants were excluded if born > 32 weeks, if they had a congenital anomaly, or if their parents did not speak English. Infants were later excluded if they became a ward of the state or if transferred to another hospital. The first 30 preterm infants were enrolled in a pilot and feasibility study in which all infants received the SENSE program. Prior to this pilot and feasibility study, the SENSE program doses had been finalized based on the systematic and scientific process used for its development. However, during the course of this pilot and feasibility study, education materials were refined based on parent feedback, observed challenges, and the need for more clarification across the protocol. Then, 70 additional preterm infants, hospitalized in private NICU rooms, were enrolled and randomized to either the SENSE program or standard-of-care. The intervention across the pilot and randomized clinical trial did not change, however, there was refinement of the educational process from pilot to randomized clinical trial. For the randomized clinical trial, 31 infants were randomized to the SENSE program, and these along with the 30 infants enrolled in the pilot and feasibility study made up the group of 61 infants used to define adoption and feasibility, time and cost, and acceptability. Fidelity was assessed comparing those who were randomized to the SENSE program (*n* = 31) compared to standard-of-care (*n* = 39).

### Overview of study procedures

Implementation of the SENSE program described in this manuscript was part of a research study. Study procedures were conducted by the research team, with the neonatal therapist who conducted the SENSE education and activated the sensory support team being a member of the research team and not part of the clinical team. For the 61 infants receiving the SENSE program, parent education occurred as soon after birth as possible (see below under ‘SENSE program education’) with additional education targeted at least weekly. The 39 infants who were randomized to standard-of-care did not receive the SENSE program, but health care professionals conducted parent education and supported parent-infant engagement based on standard NICU practices. Sensory exposures were tracked on bedside logs. Sound was captured over a 16-h period using the Language Environmental Acquisition (LENA) device within 2 weeks of birth, at 30 and 34 weeks PMA and again just prior to discharge (between 34 weeks to 48 weeks PMA). Also prior to NICU discharge, parents completed a questionnaire to define sociodemographics as well as perceptions about participation in the study. Prior to NICU discharge, feeding performance, parent-child interaction, and neurobehavior were also assessed using standardized measures, but these outcomes are not reported in this manuscript, as the focus of this manuscript is to report implementation outcomes.

### Medical factors

Infant factors were collected to give context to the population and aid in understanding the implementation of sensory interventions among medically complex infants. Infant medical factors collected included estimated gestational age (EGA), birthweight, PMA at discharge, whether part of a multiple birth, infant sex, length of stay, presence of patent ductus arteriosus (PDA), necrotizing enterocolitis (NEC), cerebral injury (defined as grade III-IV intraventricular hemorrhage), and length of endotracheal intubation and noninvasive mechanical ventilation.

### Sociodemographic factors

Sociodemographic factors that were collected included maternal age, insurance type (public or private), race (Black/African-American or not Black/African-American), number of other siblings at home, education level (some college or no college), household income (< $25,000 or > $25,000), and maternal marital status (single or married).

### The SENSE program

The age-appropriate doses of tactile, auditory, olfactory, kinesthetic/vestibular, and visual exposures were defined in the SENSE program using a rigorous scientific process prior to enrollment [13]. Printed parent education materials were developed that included information about sensory exposure in utero, the NICU environment, how to read and respond to infant behavior, as well as recommended doses of different sensory exposures across PMA and how to conduct them. The education materials were developed and validated at an eighth grade reading level to enable comprehension across the parent population.

#### Education

Parents with infants receiving the SENSE program were given initial education as soon after birth as possible by a neonatal therapist who was a member of the research team, using the SENSE education materials. The appropriate amount of tactile, auditory, visual, vestibular/kinesthetic, and olfactory stimulation for each infant’s PMA was defined (see Table 1). The research team member checked in with the family, targeted to occur at least each week, to assess the infant’s tolerance of the interventions, provide additional education, and update the appropriate doses of different sensory exposures. The type (on phone or by text or email; in-person in the NICU), timing (PMA), and amount (number of minutes) of education given to parents were tracked and were individually tailored based on the previous week’s log data, parent requests, parent receptiveness and/or comprehension, and observations made by the research team, as well as parent availability.

**Table 1:** Types and minimum daily doses of sensory input across PMA defined in the SENSE program

PMA	Tactile	Auditory	Olfactory	Visual	Kinesthetic/Vestibular
**23–27 weeks**	1 h kangaroo care or gentle human touch	Quiet conversations; no additive auditory exposures	Scent cloth	Lights off; < 10 lux	2 mins free movement 1x/day
**28–29 weeks**	1 h kangaroo care, gentle human touch, or blanket holding	20 mins reading, singing, or speaking to baby (45 dB)	Scent cloth	Lights off; < 10 lux	2 mins free movement 2x/day
**30–31 weeks**	1 ½ hr. kangaroo care, gentle human touch, or blanket holding	30 mins reading, singing, or speaking to baby (45 dB)	Scent cloth	Lights off; < 10 lux	2 mins free movement 2x/day
**32–33 weeks**	2 h of kangaroo, gentle human touch, blanket holding, or massage	1 ½ hr of reading, singing, speaking to baby, or playing soft music or recorded voice (45 dB)	Scent cloth	Cycled light (lights on during the day; 25–100 lux and lights off at night; < 10 lux). Avoid direct and bright lights.	2 mins free movement 3x/day; Rocking for 2–3 mins
**34–35 weeks**	3 h of kangaroo, gentle human touch, blanket holding, or massage	2 h of reading, singing, speaking to baby, or playing soft music or recorded voice (45 dB)	Close parent contact	Cycled light (described above) and avoid direct and bright lights.	2 mins free movement 8x/day; Rocking for 3–7 mins
**36+ weeks**	3 h of kangaroo, gentle human touch, blanket holding, or massage	3 h of reading, singing, speaking to baby, or playing soft music or recorded voice (45 dB)	Close parent contact	Cycled light (described above) and avoid direct and bright lights. Focus/follow face (12–18″ away).	2 mins free movement 8x/day; Rocking for 7 mins

#### Sensory support team

To enhance implementation, specifically fidelity, the sensory support team (trained volunteers) ensured doses of sensory exposures defined in the SENSE program were administered, even when parents were unable to be present. This was especially important at the study site, where parent participation has previously been described as suboptimal [18, 19]. Parents were encouraged to complete the daily sensory exposures described in the SENSE program, and the sensory support team completed them with medically stable infants when the parents were unable to be present or did not meet the doses as described. The neonatal therapist on the research team completed sensory exposures on infants who were medically fragile (< 32 weeks, poor tolerance of handling, or on respiratory support) when parents were not present.

### Standard-of-care

The standard-of-care group (*n* = 39) did not have clear sensory dose expectations across each day of hospitalization as detailed in the SENSE program. However, infants received sensory exposures that were part of standard-of-care. Education occurred in the context of usual care. Generally at the study site, parents were educated by nurses and neonatal therapists and encouraged to engage in skin-to-skin care and holding of their infants. This was encouraged for infants with complex medical interventions that include endotracheal intubation, but not for infants with chest tubes or on high frequency oscillatory ventilation. Reading to infants was just beginning to gain acceptance as a standard-of-care at the time of this study, but was not being used routinely.

### Tracking sensory exposures

The amount and type of sensory exposures across each weekday (5 days per week) were tracked using bedside logs (see Appendix 1 for example). The weekend was not tracked, due to lack of consistent study team presence to provide and document observed sensory exposures. For infants who received the SENSE program, the log sheets that are part of the SENSE program were used. These log sheets included the targeted amount of each sensory exposure each day, along with space for documentation of who completed the sensory exposure, which type of sensory exposure was completed, and how long the sensory exposure lasted. For infants in the standard-of-care group, log sheets included space to document sensory exposures done with the infant, but the dose target was not identified. Logs were completed by parents, the sensory support team, and health care professionals and supplemented by a member of the research team when sensory exposures were observed, but not documented. The electronic medical record was also used to supplement the amount of holding and skin-to-skin care, which contained routine documentation by medical staff. Time periods when the SENSE program was modified or put on hold were also tracked, along with the reasoning for why the program was being held.

Each week the total time and the proportion of auditory and tactile targets conducted by parents, health care professionals, and the sensory support team (amount provided divided by the total amount recommended in the SENSE program for that PMA) was calculated. Weekly percentages were averaged for a total proportion of the tactile and auditory doses conducted across the length of hospitalization. Auditory and tactile exposures were the focus, as they have large, well-defined, and discreet dose targets, in comparison to targets based on other senses, such as olfactory inputs, in which the use of a scent cloth is not easily captured as a dose (as it is used continuously) or kinesthetic, in which the dose target is a small fraction of that for auditory and tactile exposures. We defined the total number of minutes of auditory and tactile exposure, the amount of parent participation (proportion of the auditory and sensory exposures parents conducted over the length of stay); who conducted most of the SENSE interventions (the proportion of the total auditory and tactile doses in the SENSE program that parents conducted, compared to the proportion that the sensory support team and medical team conducted); and the total proportion of the SENSE auditory and tactile dose targets the infant received across hospitalization.

Sound in the infant’s bedspace was also tracked using the LENA device within 2 weeks of birth, at 30 and 34 weeks PMA and again between 34 and 48 weeks PMA, with the recording starting between 8 am-1 pm. The LENA has been used to assess the NICU environment in preterm infants [20] and measures meaningful and distant language, silence, noise, TV or electronic sounds, and the number of adult words over a 16-h period.

### Parent and family presence

The number of weekdays parents were present, the average length of each parent visit, the number of weekdays that other family visited, and the average length of each visit by other family were also tracked. In addition, the amount of time spent doing SENSE program parent education was tracked and put in context of cost.

### Parent perceptions of the SENSE program

Prior to NICU discharge, parents completed a discharge questionnaire that asked questions aimed at determining parent acceptability. Refer to Appendix 2 for survey questions.

### Implementation outcomes and analysis plan

#### Reach

Descriptive statistics were used to determine reach by defining the proportion of parents who enrolled in the study (number approached divided by number who enrolled) and defining who conducted the majority of the sensory interventions in the SENSE program over the hospitalization (parents, sensory support team, or medical team).

#### Effectiveness

Effectiveness of the SENSE program in improving infant outcomes will be reported in a separate publication. According to the RE-AIM framework, effectiveness can also include parent perceptions of the SENSE program (acceptability). Acceptability was reported on the survey contained in the discharge questionnaire. Negative outcomes, including the number of families who withdrew from the study, negative perceptions voiced by parents or health care professionals, and consequences of participation were identified. Times that SENSE program interventions were placed on hold or adapted were documented and put in context of concurrent medical factors. Specific concerns raised by the health care team, and what the research team did to address those concerns, were also tracked. Descriptive statistics were used to report parent perceptions of the SENSE program from questionnaire responses. Qualitative responses were organized into themes by an independent coder. The survey responses were then organized into each theme. Five members of the research team discussed the themes, and the comments were placed under each theme until consensus was reached (with one member of the research team consisting of an expert in the SENSE program, author RP). All comments were then organized under their themes for logical reporting. Narrative text is used to identify the number of times that the SENSE program was put on hold or adapted, along with reasons reported in addition to concerns raised by the health care team during the course of the study.

#### Adoption

To determine adoption, we used descriptive statistics to define the PMA and chronological weeks of age at SENSE program initiation, the total number of weeks each infant engaged in the SENSE program, and the proportion of families who withdrew from the study following enrollment (number of families who withdrew divided by the number of families enrolled in both the SENSE group and standard-of-care group). We investigated the relationships of timing of education (how many weeks old the infant was at initial education) and amount of parent participation (percentage of the auditory and sensory exposures parents conducted over the length of stay); amount of education (total number of minutes of education) and amount of parent participation (proportion of auditory and tactile doses conducted by parents); and total time of initial education (in minutes) and parent participation (proportion of auditory and tactile doses conducted by parents). The relationships of timing of education and amount of education with parent participation was investigated using linear regression models, with α = 0.05.

#### Implementation

We used descriptive statistics to define the total amount and proportion (amount divided by the total amount recommended in the SENSE program for that PMA) of auditory and tactile dose targets conducted by parents, health care professionals, and the sensory support team. Weekly percentages were averaged for a total proportion of the tactile and auditory doses conducted across the length of hospitalization. Descriptive statistics were used to report LENA auditory exposures across groups, and independent samples t tests were used to determine differences in auditory exposures among the SENSE and standard-of-care groups. In addition, cost was estimated as a reflection of time spent by the neonatal therapist research team member on parent education. As the education was conducted by the research team, and not part of clinical care, cost was a reflection of the amount of education provided directly to the parents of infants receiving the SENSE program. The amount of time (in minutes) of the initial education session as well as the total education time across hospitalization was calculated for each infant. Time spent on education was averaged and cost estimated on salary estimates for a neonatal therapist in the Midwest to derive a total cost per family. Of note, time assessing infants for their tolerance of the SENSE interventions or the amount of time that the neonatal therapist research team member provided interventions to medically unstable infants was not captured as part of this study. Other costs not captured included the education, training, and organization of the sensory support team. Differences in documented tactile and auditory exposures across the SENSE and standard-of-care groups were investigated using independent samples t-tests with α = 0.05) to determine treatment differentiation.

#### Maintenance

Maintenance was not assessed as part of this study, however, information learned informed future strategy for implementation across sites where maintenance can be evaluated.

## Results

Of 100 enrolled infants, 61 received the SENSE program (30 in pilot and feasibility study and 31 randomized to the SENSE group), and 39 received standard-of-care. Four infants (2 SENSE group and 2 standard-of-care group) expired. Six parents (6%), 3 in the SENSE group and 3 in the standard-of-care group, withdrew from the study. Three did not specify reasoning for withdrawal, one was withdrawn due to discovery of a congenital anomaly (which was an exclusion criteria), one became a ward of the state, and one family withdrew following illness leading to isolation. Nine infants (9%) were transferred to another hospital prior to the completion of all study procedures, and therefore were excluded due to missing information. Data from 81 parent-infant dyads (52 who received the SENSE program and 29 who received standard-of-care and did not receive the SENSE program) were included in analyses.

### Reach

There were 131 families recruited, and 100 enrolled (76%). Reasons for declining included lack of interest in participating in research (*n* = 4), lack of interest in participating (*n* = 6), feeling overwhelmed (*n* = 5), worried about volunteers interacting with baby (*n* = 1), mother was interested but father was not (*n* = 2), transfer to another hospital after being approached (*n* = 1), and failure to follow through with paperwork within the enrollment window (*n* = 2).

Among infants who received the SENSE program, the majority (*n* = 44/52; 85%) had most of their sensory interventions performed by parents, whereas 6/52 (12%) had most performed by the sensory support team, and 2/52 (4%) by the medical team.

### Effectiveness

Seventy-one (88%) of parents completed the survey. Refer to Table 2 for descriptives on parent survey responses. Refer to Appendix 3 (Table 8 and Table 9) for additional comments made by parents who did and did not receive the SENSE program.

**Table 2:** Survey responses from parents of infants who received and did not receive the SENSE program

**Survey Questions:** 0-Very unsatisfied; 100-Very satisfied	**Standard-of-care group**	**SENSE group**	*p **value**
	*N* = 19	*N* = 52	
How satisfied were you with the SENSE study?	78.1 (16.5)	82.0 (30.5)	0.60
Would you recommend the study to other parents with babies in the NICU?	87.8 (14.5)	95.5 (11.9)	**0.02**
Did the study help you to learn how to provide sensory experiences for your child?	0.8 (0.4)	100 (0)	**0.04**
How much did the study help you. Become more comfortable in the NICU with your child?	56.4 (31.2)	91.8 (15.7)	**< 0.0001**
How much did the study help you be a parent for your child in the NICU?	55.4 (29.9)	85.7 (19.5)	**< 0.0001**
How much did the study help you when you had to be away from your child in the NICU?	49.8 (37.2)	84.2 (23.0)	**0.002**
How much did the study help your child?	66.4 (33.4)	89.5 (16.8)	**0.01**
What were your main worries about joining the study?			
Worries: I wasn’t sure how it worked	6 (27%)	13 (27%)	0.95
Worries: I was worried about someone else holding my baby	4 (18%)	5 (10%)	0.35
Worries: I was worried about the volunteers	4 (18%)	1 (2%)	**0.01**
Worries: I didn’t know if my baby really needed it	1 (5%)	3 (6%)	0.79
Worries: I did not have any worries	11 (50%)	35 (71%)	0.08

*P value is from investigating differences in responses using independent samples t-tests. Bold values are those that reached significance (*p* < 0.05), indicating a difference in the standard-of-care and SENSE groups

During the SENSE program implementation (pilot and feasibility study), health care professionals were largely supportive of the SENSE program tasks. Two main themes of concern emerged by health care professionals during the course of the study: overstimulation and timing of interventions. Concerns about overstimulation were addressed through parent education on determining infant readiness for stimulation based on physiological and behavioral cues as well as by instituting assessments to determine tolerance of the described sensory exposures. This aided in ruling-out overstimulation and ensured all the interventions were done in a way that respected the infant’s behavioral cues. The other theme that emerged was the timing of interventions. Current models in the NICU support the use of clustered care [21], where multiple interventions are done clustered together in close proximity to a care time to allow the infant to have long periods of uninterrupted sleep in between care times. However, the positive sensory exposures did not always occur around a clustered care time. For example, gentle human touch may be applied halfway between cares to aid the infant in settling into sleep, or the parent may choose to hold for a long period after a feeding. This concern enabled refinement of the educational materials to define what activities could be done to aid sleep (skin-to-skin, gentle human touch, music) and what activities could rouse the infant and should be done clustered around a care time (massage, transfer out of bed). These changes were clarified with health care professional staff, leading to acceptability thereafter. One additional concern identified by the research team was the role of the sensory support team (volunteers). The initial intent was for the sensory support team to engage in holding, reading, playing music, and gentle human touch. Due to lack of full confidence in volunteers providing interventions with a range of different infants during the pilot and feasibility study, their role was redefined to include low-risk tasks of providing gentle human touch and reading to the infant.

Seventy-seven infants (95%) tolerated the SENSE program interventions and dose targets as defined. Four infants (5%) had documentation of intermittent periods when the SENSE program had to be put on hold or modified due to lack of infant tolerance. Lack of tolerance was related to these infants progressing to PDA ligation, having physiological instability with high frequency oscillatory ventilation, hernia repair, as well as end of life discussion and subsequent tracheostomy. All of these infants had actively engaged parents and were able to meet the dose targets of auditory and tactile exposures over the length of stay, despite the average time for stopping or modifying interventions being 1–2 weeks.

### Adoption

Refer to Table 3 for characteristics of the 81 infants in the sample. See Table 4 for descriptives on the amount of SENSE program education provided to parents. Infants received 3–20 weeks of the SENSE program, with an average of 10.1 (±7.6) weeks. Earlier timing of initial education (weeks of age) was related to more parent participation (proportion of auditory and tactile exposure targets done by parents); *p* = 0.049, Beta = 10.3. Parent participation was not related to number of educational sessions (*p* = 0.51), total time of education (*p* = 0.54), PMA at the time of education (*p* = 0.59), or amount of time spent on initial education (*p* = 0.058).

**Table 3:** Sample characteristics

Factor (*n* = 81, unless otherwise specified)	Total Mean ± SD, N (%), or Median (IQR)	Standard-of-care group Mean ± SD, N (%), or Median (IQR)	SENSE group Mean ± SD, N (%), or Median (IQR)	*p value (Differences between groups)
Medical Factors				
EGA at birth (weeks)	29.2 ± 2.5	29.5 ± 2.5	29.1 ± 2.6	0.44
Birthweight (g)	1359.9 ± 463.4	1416.2 ± 477.8	1328.5 ± 456.8	0.42
PMA at discharge (weeks)	39.8 ± 7.1	39.8 ± 5.8	39.8 ± 7.8	0.98
Multiple birth	29 (36%)	13 (45%)	16 (31%)	0.21
Infant sex (male)	32 (40%)	11 (38%)	21 (40%)	0.83
Length of stay (days)	76.8 ± 57.5	74.7 ± 54.7	77.9 ± 59.4	0.81
Presence of PDA	19 (23%)	6 (21%)	13 (25%)	0.66
Presence of NEC *n* = 80	4 (5%)	2 (7%)	2 (4%)	0.56
Presence of PVL	4 (5%)	1 (3%)	3 (6%)	0.64
Presence of IVH	14 (17%)	3 (10%)	11 (21%)	0.22
# days on mechanical ventilation *N* = 79	1 (0–6)	1 (0–7.8)	1 (0–4)	0.70
# Days on non-invasive mechanical ventilation (CPAP/SiPAP) *n* = 79	6 (2–18.3)	5 (2–19)	6 (2–18)	0.88
Sociodemographics				
Maternal age (years)	28.2 ± 6.3	30.9 ± 7.2	26.6 ± 5.3	**0.003**
Insurance type (public)	55 (68%)	18 (62%)	37 (71%)	0.40
Infant race (Black/African-American)	36 (44%)	10 (34%)	26 (50%)	0.39
# of siblings	1.5 ± 1.0	1.7 ± 2.1	1.5 ± 1.6	0.58
Education level (some college or more) *n* = 75	60 (80%)	18 (78%)	42 (81%)	0.31
Income level (< $25,000/year) *n* = 75	34 (45%)	7 (30%)	27 (52%)	0.41
Maternal marital status (married) *n* = 77	31 (40%)	13 (52%)	18 (35%)	0.41

*p value is from investigating differences across those who did and did not receive the SENSE program using independent samples t-tests and chi-square analyses. Bold values are those that reached significance (*p* < 0.05), indicating a difference in the standard-of-care and SENSE groups

**Table 4:** SENSE program education descriptives

*N* = 52	n (%)	Min-Max (range)	Mean (SD) or n (%)
Families received education	49 (94%)		
Total time on parent education, minutes		15–150	72.3 (37.4)
Number of education sessions		0–18	4.8 (3.7)
Time spent on education per session, minutes		2–25	9.2 (4.5)
Time on initial education, minutes		5–75	30.4 (16.0)
PMA when education started		23–34	29.8 (2.4)
Week of life education started		0–3	0.7 (0.8)
Education received by phone/email; total time on phone/email education, minutes	24 (49%)	1–86	24.3 (25.4)
Education received in-person; total time spent on in-person education, minutes	36 (73%)	10–130	41.7 (28.7)

There were 6/100 (6%) families who withdrew from the study, but none of these withdrawals were related to concerns about the SENSE program.

### Implementation

See Table 5 for the amount of tactile and auditory exposures infants in the SENSE group received across hospitalization. Among infants randomized to standard-of-care or SENSE programming, there was a significant difference in the proportion of tactile and auditory exposures identified in the SENSE program that the infant received; the mean proportion of tactile and auditory target doses (defined in the SENSE program) that were met by parents, health care professionals, and the sensory support team was 91% in the SENSE group compared to 48% in the standard-of-care group, *p* < 0.0001. See Table 6.

**Table 5:** Auditory and tactile sensory exposures administered across hospitalization among infants receiving the SENSE program

Type of sensory exposure	Total number of minutes done by parents across hospitalization	Total number of minutes done by medical team across hospitalization	Total number of minutes done by sensory support team across hospitalization	Total number of minutes done by parents, medical team and support team combined across hospitalization
**Tactile** (skin-to-skin, holding, massage, gentle human touch)	**Median (IQR)**			
	4730 (2165-8025)	1120 (525–1739)	730 (499–1440)	5138 (3075-9278)
	**Range (Min-Max)**			
	515–28,755	215–4805	20–4230	665–29,760
**Auditory** (reading, singing, talking to; playing recorded sound or music)	**Median (IQR)**			
	3398 (1729-4828)	455 (146–1133)	1020 (510–2048)	4018 (2104-6383)
	**Range (Min-Max)**			
	335–13,800	80–1680	120–5080	560–14,040
**Tactile & Auditory Combined**	**Median (IQR)**			
	7900 (3615-12,701)	1153 (570–2359)	1720 (945–3591)	9325 (5295-15,694)
	**Range (Min-Max)**			
	850–42,555	290–5805	20–9310	1225-43,800

**Table 6:** Differences in SENSE program doses achieved among the SENSE group and standard-of-care group

	SENSE group	Standard-of-care group	Mean difference	*p value
Mean (SD) Proportion of Tactile and Auditory Doses Done by Parents	69.9 (29.0)	47.7 (29.2)	22.2	0.001
Mean (SD) Proportion of Tactile and Auditory Doses Done by Parents, Health Care Professionals and Sensory Support Team	91.0 (17.6)	47.9 (28.9)	43.0	< 0.0001

*p value is from investigating differences in those who did and did not receive the SENSE program using independent samples t-tests

In the control group, 10% (*n* = 3/29) of infants received more than 100% of the auditory and tactile doses defined in the SENSE program. In the SENSE group, 72% (*n* = 36/50) received greater than 100% of the auditory and tactile doses defined in the SENSE program. There was a significant difference in the proportion of the auditory and tactile dose targets achieved between groups (*p* < 0.0001).

In the control group, 7/29 (24%) infants received > 75% of the auditory and tactile doses in the SENSE program compared to 44/52 (85%) in the SENSE group. There was a significant difference between groups (*p* < 0.0001). The SENSE group had an average of 14 min and 56 s more meaningful language than the standard-of-care group in a 16-h period on the LENA (*p* = 0.01).

Eight infants (7%) who received the SENSE program did not receive the recommended doses of auditory and tactile dose targets defined in the SENSE program.

Among 61 infants that had parent presence and holding tracked, parents were present an average of 3.8 (±1.3) days per 5-day week for an average of 6.3 (±4.3) hours per day. Other family visited 1.3 (±1.3) days per 5-day week. Parents held their infants for 3.3 (±1.5) days per 5 day week and did skin-to-skin 1.5 (±1.9) days per 5-day week for an average of 2.7 (±3.3) hours per day. The average number of days the infant was held per 5-day week was 3.3 (±1.5), and the average number of days the infant was held skin-to-skin was 1.5 (±1.6) days per 5-day week. No differences between the raw amount of recorded parent presence, holding, or skin-to-skin (not cumulative tactile total) in those who did and did not receive the SENSE program were observed.

See Table 7 for auditory descriptives, with more meaningful language observed among SENSE recipients (*p* = 0.013).

**Table 7:** Descriptives of auditory exposures on the LENA across the SENSE group and standard-of-care group

	Standard-of-care group (*n* = 23)	SENSE group (*n* = 35)	Mean Difference	95% Confidence Internal of the Difference	*p* value^*^
	Mean (h:min) ± SD	Mean (h:min) ± SD	(h:min)	(h:min)	
Amount of time spent with meaningful language	0:16 **±** 0:16	0:31 **±** 0:27	−0:14	[−0:26, −0:03]	**.013**
Amount of time spent with distant language	0:44 **±** 0:37	0:58 **±** 1:03	−0:13	[− 0:40, 0:12]	.307
Amount of time spent with TV/electronic sounds	2:01 **±** 2:09	2:44 **±** 2:58	− 0:42	[−2:03, 0:38]	.294
Amount of time spent with noise	4:21 **±** 5:00	2:39 **±** 3:39	1:41	[1:12, −0:45]	.170
Amount of time spent in silence	8:35 **±** 4:33	9:06 **±** 3:46	−0:30	[1:08, −2:49]	.660
Number of spoken words around the infant	2919 **±** 3726	3613 **±** 4438	−694	[− 2876, 1487]	.526

*p value is from investigating differences across the SENSE and standard-of-care groups using independent samples t-tests after log transformation of LENA outcome measures. Bold values are those that reached significance (*p* < 0.05), indicating a difference in the standard-of-care and SENSE groups

See Table 4 for descriptives related to SENSE program education for those who received the SENSE program. The average total time spent on education was 72.3 min (±37.4). The average salary of an occupational therapist in Missouri is approximately $79,570 based on data from US Bureau of Labor Statistics [22]. When an additional 20% is added to this figure as an estimate of fringe benefits, the cost of a neonatal therapist in this study setting is approximately $95,484 for a full-time equivalent position. Estimating 40 h per week for this salary cost would make the hourly cost of the personnel to do the SENSE program education be approximately $45.90 per hour. At this cost, education of one set of parents across hospitalization to conduct the SENSE program would cost $57.38. It would be anticipated that these costs of education could be encapsulated in existing neonatal therapy programming, and if so, would not represent a true cost to the hospital, but would be billable, charged under neonatal therapy services using an average charge of $200 per hour [23], with billable charges of an average total of $250 per participant (average of 73 min of total education @ $200/h = $250). Using this estimated billing minus the cost of the therapist would result in costs that were recuperated through billing.

## Discussion

The key findings of this study were that the SENSE program, implemented within a research study, was able to reach > 75% of the targeted population with < 10% attrition. Parent feedback was largely positive about participation in the study. The total education time provided to parents was an average of 73 min. Large amounts of positive sensory exposures [a median of 9325 (5295-15,694) minutes of auditory and tactile exposures] were achieved across hospitlization, and differences in sensory exposures among those who did and did not receive the SENSE program were evident. Eighty-five percent of parents who received the SENSE program conducted the majority of the SENSE interventions, and when combined with the sensory support team, 91% of infants received all of the sensory experiences defined in the SENSE program across hospitalization.

The SENSE program was feasible to implement with preterm infants across hospitalization using a research team to faciliatate programming. Use of the SENSE program led to more positive sensory exposures across hospitalization. Ninety-five percent of infants in a Level IV NICU could tolerate the SENSE program exposures as defined, and only 5% required occasional periods of modification or stopping interventions for medical instability. Of interest was that the earlier the SENSE program education and interventions occurred, the more parent engagement occurred. To our knowledge, we are the first study to report that earlier engagement leads to better parent participation in the NICU. Ninety-one percent of the auditory and tactile dose targets defined in the SENSE program occurred among those receiving the SENSE program. Seventy two percent of infants received more than the dose targets described in the SENSE program. While 3 h of auditory and 3 h of tactile exposures may sound like a large amount of additional sensory exposures to accomplish, families were able to find ways to incorporate them into daily life in the NICU to mitigate painful or procedural sensory experiences.

Parents reported high acceptability. Previous work identified a subset of parents who voiced concerns about other personnel interacting with their infant, however, having health care professionals and/or the sensory support team nurture and provide extra attention to their infant in their absence was viewed as a positive factor among most parents who were surveyed. This parallels an increase in reports of NICU volunteers [24, 25], who can serve as sensory support team members. While some parents commented that logging their activities increased awareness of what they could do with their infant(s), most parents reported the bedside logs as something they disliked about the study. This prompts motivation for the development of innovative ways to aid parents and the sensory support team in tracking sensory exposures. Finally, parents reported that the SENSE program helped them build confidence interacting with their child, which is consistent with previous pilot findings demonstrating relationships between the SENSE program and parent confidence [26].

SENSE program education was an average of 73 min, which can be recuperated in standard billing by a neonatal therapist. The American Academy of Pediatrics recommends that Level III and IV NICUs have an occupational or physical therapist on staff [27], and the role of SENSE administrator (taken on by the occupational and physical therapist as part of the research team for this study) could easily be adopted by the neonatal therapist and included in typical neonatal therapy services. While the general cost of programming is neglible, training, organizing, and supervising a sensory support team to carry out sensory exposures when parents are unable to may add additional costs that are not captured in this manuscript.

There were limitations to this study. The sample size was pre-determined based on the 2 overarching cohorts, and power analysis was not conducted on implementation outcomes to ensure adequate sample size. Most of the outcomes are descriptive in nature. Enrollment in a study that is investigating different types and quantities of sensory exposures suggests that this is what parents should be doing and could also have impacted parent behavior. Log sheets used to track sensory exposures also are suggestive, even though the doses were not listed on the standard-of-care group logs, which could have led to more parent engagement due to power of suggestion. Despite these limitations, we were able to demonstrate significant differences in the proportion of auditory and tactile exposures completed with infants between the SENSE program group and standard-of-care group. This study was also limited by error introduced by logging and reporting of sensory exposures across multiple sources. Twenty-four hour videorecording was attempted to better capture the sensory environment, but was not feasible. Other limitations include subjective interpretation of survey questions and parent’s answers, lack of capture of other costs that were related to implementation (such as managing the sensory support team and conducting sensory experiences on medically unstable infants when parents were unavailable), and lack of capture of other sensory exposures (vestibular/kinesthetic, olfactory, visual) when investigating fidelity. This manuscript includes a very large amount of data on sensory exposures across long periods of hospitalization, which increases the risk of error and/or omission. In addition, the large amount of data is managed by use of descriptive analysis, of which average or median values may not provide the same level of understanding as individual reports on the application of sensory exposures in the moment. There are also a multitude of implementation factors that can be investigated, and this study only reports on a few. However, we were able to use a systematic process to report implementation outcomes using the RE-AIM framework to speed the translation of research findings to the bedside.

## Conclusion

The SENSE program is an implementation strategy designed to ensure that appropriate positive sensory exposures are delivered to high-risk infants each day of NICU hospitalization in order to improve their health care experience and drive appropriate brain development. This study reports on implementation within the context of a research study, with research team personnel used to administer the SENSE program in a level IV NICU. Refinement of educational materials for parents as well as implementation materials for hospitals was undertaken, using knowledge gained from this study. Following this study, there was increased interest across the United States and abroad in the SENSE program, and care was taken to define a dissemination and implementation strategy to aid its uptake across different hospitals. Given that the study had a research team member administer the program, one important component of the implementation strategy that was detailed and included with SENSE program educational materials for distribution was the identification of a SENSE administrator at each site. This SENSE administrator oversees the education of parents, assesses tolerance of the infants, and provides access to program materials. The use of a facilitator, or SENSE administrator, is in line with frameworks that support the use of facilitation [28, 29]. The facilitator, or SENSE administrator, optimizes the adoption of the SENSE program by tailoring it to the local hospital setting, engaging in interactive problem solving, and promoting the success of the program [30]. In doing so, the administrator puts the evidence-based SENSE program into the context of the NICU, considering policies and procedures, level of acuity, and culture and communication. By using existing personnel, such as a neonatal therapist, to administer the SENSE program, additional costs can be avoided. Further, use of volunteers or Cuddler programs within each hospital setting can enable additional sensory support for infants when parents are unable to be present. With high demand of over 230 hospitals implementing the SENSE program as of February 2021, further investigation of implementation by clinicians, rather than by a research team, is warranted. Future work that investigates multiple-site implementation and enhanced strategies for implementation at the organizational level and individual level will aid in uptake. Finally, investigating maintenance (from the RE-AIM framework) is an important area of research as uptake across other hospitals occurs. In regards to the SENSE program itself, a critical component is that it remain rooted in current evidence. It will not remain a static intervention across time, as it will be updated, informed by new evidence that does not yet exist. We have carefully operationalized implementation outcome definitions here to enable replication on the current and/or future SENSE programming.

## Data Availability

De-identified data and materials used for this study will be made available to other researchers upon request.

## Appendix 2

### Survey questions to determine parent acceptability

1. Overall, how satisfied were you with the SENSE study?
  0 (Very Unsatisfied) - 100 (Very Satisfied)
2. Would you recommend the study to other parents with babies in the NICU?
  0 (Very Unsatisfied) - 100 (Very Satisfied)
3. Did the study help you to learn how to provide sensory experiences for your child?
  0 (Very Unsatisfied) - 100 (Very Satisfied)
4. How much did the study help you become more comfortable in the NICU with your child?
  0 (Very Unsatisfied) - 100 (Very Satisfied)
5. How much did the study help you be a parent for your child in the NICU?
  0 (Very Unsatisfied) - 100 (Very Satisfied)
6. How much did the study help you when you had to be away from your child in the NICU?
  0 (Very Unsatisfied) - 100 (Very Satisfied)
7. How much did the study help your child?
  0 (Very Unsatisfied) - 100 (Very Satisfied)
8. What were your main worries about joining the study?
  (I wasn't sure how it worked; I was worried about someone else holding my baby; I was worried about the volunteers; I didnt know if my baby really needed it; No worries; Other)
9. What did you like about being in the SENSE study? Open ended
10. What did you not like about being in the SENSE study? Open ended

## Appendix 3

**Table 8:** Open-ended survey responses from parents of infants who received and did not receive the SENSE program, stating what they liked about their involvement in the research study

What did you like about the study?		
***Themes***	Comments from those who received the SENSE program	Comments from those who did not receive the SENSE program
***Sensory support team/Human and nurturing presence***	“That there was always someone there with my babies.” “I loved that my baby got extra attention and care in my absence.” “The attention my daughter got when I wasn’t here.” “Everything they did and the help provided for my daughter.” “Seeing the girls every day! Having someone available when we weren’t there!” “Good support.” “My baby had visits when in between my visits. Someone was spending time with him, talking and singing to him while I was gone. He was not alone.” “All the support from the SENSE team.” “My baby had interactions when I couldn’t be here.” “That the days when we weren’t available to come and see Jacob, the women from the SENSE study came to visit. Thank you.” “They came in and did comfort touch with the baby.” “Someone was there to comfort my babies when I wasn’t there.” “The volunteers.” “I loved knowing that someone could show my child that they weren’t alone when I wasn’t able to be here.” “When I wasn’t able to be at the hospital someone was there with him.” “I like that someone came around to check on me and my baby.” “I like that people from the research study stop by [Rebecca’s] bedside often to see how she and I were doing.” “Constant visits.” “I liked the staff”	“My baby always being looked after.” “Gave my baby the extra care and support she needed.” “Very nice and friendly people.”
***Educational materials***	“The study was great! I loved having the binder that gave me developmental-appropriate activities to engage with my baby.” “I liked the reading materials” “If I needed more books they were willing to get me more.” “How it only takes one little device [log sheets] to track things in a little person.”	“Books.” “Products helping support the babies (booklet)”.
***Parent education***	“They taught me how to recognize when my child was stressed.” “I liked all the neat things that you learned about.” “The information.” “The study taught me how to help my baby advance by the week. Ex: when and how long to read, hold, rock and play music to him.” “It gave us awareness about a lot of things that come with dealing with a premature baby. It made us pay attention to our stays with having to log what we do. Even though we missed logging a couple of days.” “I like that it guided me to do different things with my baby like reading, singing, and holding my baby.” “It showed me how to provide more care for my daughter” “It gave me practical ways to help and bond with [Jimmy]”	“The tips I got for my baby’s needs. To really see how much we’ve grown in the NICU.” “Learning new things.” “Helped me help my twins.” “It was nice to track when I was in the NICU, and see what activities I could do with my baby.” “Knowing if interaction with a preemie helped or hurt the baby as opposed to just letting him sleep and grow.” “It made me more aware of the sensory activities I could share with my baby.” “Filling out the questionnaire daily kept me aware of how I was spending my time with my babies and made me think about beneficial ways to spend that time.”
***Parent/child Interaction***	“It gave me a much better idea on how to interact and be positive with my kids.” “Interaction with my baby.” “The timing of holding him.” “It gave us more time with holding our son.” “[Abby] needed constant care, contact and stimuli.” “Help with caring for my children through touch.” “Got me more involved.” “I liked the closeness I got with my kids. “It gave me confidence to be involved.”	
***Participation in research***	“Helping to expand research to help other babies in the NICU.” “I like that we can hopefully impact other lives with this study.” “I enjoyed sharing our experience and the development that came as a result.” “Gift card at the end.”	“Just liked what they studied.” “How they informed about the study.” “I know it’s very important for parents to be with their babies in the NICU so I am glad the study is trying to make things better for them.”
***Developmental impact***	“They did great exercises and all types of activities with my baby that were very helpful.” “It helped give my baby a well-rounded experience even though she could not be home.”	“Getting to see my child grow and get stronger.” “Being able to share info about progression.” “Towards the end they evaluated his developmental skills.” “Seeing the evaluation toward the end of the hospital stay.”

**Table 9:** Open-ended survey responses from parents of infants who received and did not receive the SENSE program, stating what they did not like about their involvement in the research study

What did you not like about the study?		
***Theme***	Comments from those who received the SENSE program	Comments from those who did not receive the SENSE program
***Staff related/ Communication***	“I frequently missed the volunteers during the day.” “Need more staff to help after hours so babies get more time all day.” “Communication there wasn’t much communication between the study and mom.”	“Not knowing when they were coming to my kiddos room.” “No communication, really didn’t know the outcome.” “We hardly talked to or seen anyone from the study.”
***Videorecording***	“Being recorded, but you forget it’s there after a while.” “Not being able to get a copy of recordings.”	“The cameras.” “Recording.”
***Paperwork/ Logging***	“Having the record everyday.” “The log, my baby needed me more and more, I did not have time to keep track of the activities.” “I’m not going to say it was a dislike, but it was a little hard writing in the book every day. Some days I would forget because my main focus was my baby.”	“I was primarily recording our time spent in the NICU.” “Keeping track of how time was spent was a challenge some days when there was more than just me visiting.” “Often forgetting to fill out his paperwork weekly.” “Too much paper work!” “All the paperwork.” “Filling out the questionnaires--way too long with redundant questions.” “Remembering to fill out the binder.”
***Other***	“On my part, not being able to do more, due to limited time or distraction at the NICU.”	“I didn’t feel like there was a learning aspect to it.” “… wasn’t given many resources to learn the benefits of different activities done with the babies.”
***Nothing***	“Nothing” responses × 27 “Nothing, I really liked the study.” “Nothing! Everyone was absolutely wonderful!” “There’s not anything that I disliked about the study. Me and my baby have a great bond because of the study.”	“Nothing” × 4

## Abbreviations

NICU: Neonatal intensive care unit
SENSE: Supporting and Enhancing NICU Sensory Experiences
PMA: Postmenstrual age
RE-AIM framework: Reach, Effectiveness, Adoption, Implementation, Maintenance framework
EGA: Estimated gestational age
PDA: Patent ductus arteriosus
NEC: Necrotizing enterocolitis

## References

[1] Pineda R, Guth R, Herring A, Reynolds L, Oberle S, Smith J (2017). Enhancing sensory experiences for very preterm infants in the NICU: an integrative review. J Perinatol.

[2] Feldman R, Rosenthal Z, Eidelman AI (2014). Maternal-preterm skin-to-skin contact enhances child physiologic organization and cognitive control across the first 10 years of life. Biol Psychiatry.

[3] Procianoy RS, Mendes EW, Silveira RC (2010). Massage therapy improves neurodevelopment outcome at two years corrected age for very low birth weight infants. Early Hum Dev.

[4] Juneau AL, Aita M, Heon M (2015). Review and critical analysis of massage studies for term and preterm infants. Neonatal Netw.

[5] Lawhon G, Hedlund RE (2008). Newborn individualized developmental care and assessment program training and education. J Perinat Neonatal Nurs.

[6] The Cope NICU program: an evidenced-based educational-behavioural intervention program for parents of preterm infants: Cope for Hope; 2019. Available from: http://www.copeforhope.com/nicu.php. Accessed Sept 2020.

[7] Welch MG, Hofer MA, Brunelli SA, Stark RI, Andrews HF, Austin J (2012). Family nurture intervention (FNI): methods and treatment protocol of a randomized controlled trial in the NICU. BMC Pediatr.

[8] Fox MK, Pac S, Devaney B, Jankowski L (2004). Feeding infants and toddlers study: What foods are infants and toddlers eating?. J Am Diet Assoc.

[9] About FICare: Family Integrated Care; 2019. Available from: familyintegratedcare.com/about-ficare. Accessed Sept 2020.

[10] The Empower Program: DandleLION Medical; 2020. Available from: www.dandlelionmedical.com/products/the-empower-program/. Accessed Sept 2020.

[11] White-Traut R, Norr KF, Fabiyi C, Rankin KM, Li Z, Liu L (2013). Mother-infant interaction improves with a developmental intervention for mother-preterm infant dyads. Infant Behav Dev.

[12] Pineda R, Roussin J, Heiny E, Smith J. Health care professionals’ perceptions about sensory-based interventions in the NICU. Am J Perinatol. 2018. 10.1055/s-0038-1676536 PMC6635089 30577058

[13] Pineda R, Raney M, Smith J (2019). Supporting and enhancing NICU sensory experiences (SENSE): defining developmentally-appropriate sensory exposures for high-risk infants. Early Hum Dev.

[14] Glasgow RE, Vogt TM, Boles SM (1999). Evaluating the public health impact of health promotion interventions: the RE-AIM framework. Am J Public Health.

[15] What Is RE-AIM: RE-AIM; 2020. Available from: https://www.re-aim.org/about/what-is-re-aim/. Accessed Sept 2020.

[16] Proctor E, Silmere H, Raghavan R, Hovmand P, Aarons G, Bunger A (2011). Outcomes for implementation research: conceptual distinctions, measurement challenges, and research agenda. Admin Pol Ment Health.

[17] Pye V, Taylor N, Clay-Williams R, Braithwaite J (2016). When is enough, enough? Understanding and solving your sample size problems in health services research. BMC Res Notes.

[18] Reynolds LC, Duncan MM, Smith GC, Mathur A, Neil J, Inder T (2013). Parental presence and holding in the neonatal intensive care unit and associations with early neurobehavior. J Perinatol.

[19] Pineda R, Bender J, Hall B, Shabosky L, Annecca A, Smith J (2018). Parent participation in the neonatal intensive care unit: predictors and relationships to neurobehavior and developmental outcomes. Early Hum Dev.

[20] Liszka L, Smith J, Mathur A, Schlaggar BL, Colditz G, Pineda R (2019). Differences in early auditory exposure across neonatal environments. Early Hum Dev.

[21] Verklan MT, Walden M (2010). Developmental support. Curriculum for neonatal intensive care nursing.

[22] Occupational Outlook Handbook: Occupational Therapists: U.S. Bureau of Labor Statistics; 2019. Available from: https://www.bls.gov/ooh/healthcare/occupational-therapists.htm#tab-7. Accessed Sept 2020.

[23] Occupational therapy cost: How much does occupational therapy cost? : Costhelper; 2020. Available from: https://health.costhelper.com/occupational-therapy.html. Accessed Sept 2020.

[24] Clubbs BH, Barnette AR, Gray N, Weiner L, Bond A, Harden J (2019). A community hospital NICU developmental care partner program: feasibility and association with decreased nurse burnout without increased infant infection rates. Adv Neonatal Care.

[25] Minear S, Wachman EM (2019). Management of newborns with prenatal opioid exposure: one institution’s journey. Clin Ther.

[26] Pineda R, Wallendorf M, Smith J (2020). A pilot study demonstrating the impact of the supporting and enhancing NICU sensory experiences (SENSE) program on the mother and infant. Early Hum Dev.

[27] Riley LSA, Kilpatrick S (2012). Guidelines for perinatal care.

[28] Harvey G, Kitson A (2016). PARIHS revisited: from heuristic to integrated framework for the successful implementation of knowledge into practice. Implement Sci.

[29] Harvey GKA (2015). Implementing evidence-based practice in healthcare: a facilitation guide.

[30] Ritchie MJDK, Miller CJ, Oliver KA, Smith JL, Lindsay JA, Kirchner JE (2017). Using implementation facilitation to improve care in the veterans health administration.

